# Adoption of guidelines on and use of oral pre-exposure prophylaxis: a global summary and forecasting study

**DOI:** 10.1016/S2352-3018(21)00127-2

**Published:** 2021-07-12

**Authors:** Robin Schaefer, Heather-Marie A Schmidt, Giovanni Ravasi, Antons Mozalevskis, Bharat B Rewari, Frank Lule, Kouadio Yeboue, Anne Brink, Nabeel Mangadan Konath, Mukta Sharma, Nicole Seguy, Joumana Hermez, Ahmed S Alaama, Naoko Ishikawa, Boniface Dongmo Nguimfack, Daniel Low-Beer, Rachel Baggaley, Shona Dalal

**Affiliations:** a Global HIV, Hepatitis and STIs Programmes, World Health Organization, Geneva, Switzerland; b UNAIDS Regional Office for Asia and the Pacific, Bangkok, Thailand; c Pan-American Health Organization, Washington, DC, USA; d World Health Organization Regional Office for Europe, Copenhagen, Denmark; e World Health Organization Regional Office for South-East Asia, New Delhi, India; f World Health Organization Regional Office for Africa, Brazzaville, Republic of the Congo; g World Health Organization Regional Office for Africa, Inter-Country Support Team, Ouagadougou, Burkina Faso; h World Health Organization Regional Office for the Western Pacific, Manila, Philippines; i World Health Organization Regional Office for the Eastern Mediterranean, Cairo, Egypt

## Abstract

**Background:**

In 2016, the UN General Assembly set a global target of 3 million oral pre-exposure prophylaxis (PrEP) users by 2020. With this target at an end, we aimed to assess global trends in the adoption of WHO PrEP recommendations into national guidelines and numbers of PrEP users, defined as people who received oral PrEP at least once in a given year, and to estimate future trajectories of PrEP use.

**Methods:**

In this global summary and forecasting study, data on adoption of WHO PrEP recommendations and numbers of PrEP users were obtained through the Global AIDS Monitoring system and WHO regional offices. Trends in these indicators for 2016–19 by region and for 2019 by country were described, including by gender and priority populations where data were available. PrEP user numbers were forecasted until 2023 by selecting countries with at least 3 years of PrEP user data as example countries in each region to represent possible future PrEP user trajectories. PrEP user growth rates observed in example countries were applied to countries in corresponding regions under different scenarios, including a COVID-19 disruption scenario with static global PrEP use in 2020.

**Findings:**

By the end of 2019, 120 (67%) of 180 countries with data had adopted the WHO PrEP recommendations into national guidelines (23 in 2019 and 30 in 2018). In 2019, there were about 626 000 PrEP users across 77 countries, including 260 000 (41·6%) in the region of the Americas and 213 000 (34·0%) in the African region; this is a 69% increase from about 370 000 PrEP users across 66 countries in 2018. Without COVID-19 disruptions, 0·9–1·1 million global PrEP users were projected by the end of 2020 and 2·4–5·3 million are projected by the end of 2023. If COVID-19 disruptions resulted in no PrEP user growth in 2020, the projected number of PrEP users in 2023 is 2·1–3·0 million.

**Interpretation:**

Widespread adoption of WHO PrEP recommendations coincided with a global increase in PrEP use. Although the 2020 global PrEP target will be missed, strong future growth in PrEP use is possible. New PrEP products could expand the PrEP user base, and, with greater expansion of oral PrEP, further adoption of WHO PrEP recommendations, and simplified delivery, PrEP could contribute to ending AIDS by 2030.

**Funding:**

Unitaid, Bill & Melinda Gates Foundation, and WHO.

## Introduction

The efficacy of oral pre-exposure prophylaxis (PrEP) with tenofovir-based antiviral medication to prevent the acquisition of HIV in uninfected people has been shown in randomised controlled trials across settings and populations.^1^ The US Food and Drug Administration approved tenofovir disoproxil fumarate with emtricitabine for oral PrEP in 2012,^2^ and, in 2015, WHO recommended offering once-daily oral PrEP to people at substantial risk of HIV acquisition (provisionally defined as HIV incidence of >3 per 100 person-years in the absence of PrEP) as an additional choice in combination HIV prevention.^3^ WHO updated guidance in 2019 to include the option of event-driven dosing (also known as on-demand PrEP) for men who have sex with men.^4^ Event-driven dosing consists of a double dose of tenofovir disoproxil fumarate with emtricitabine 2–24 h before sex, followed by one dose at 24 h and another at 48 h after the first (called 2 +1 + 1). If sexual activity continues, a single dose can be taken daily until 2 days after the last sex act. In 2021, WHO recommended the dapivirine vaginal ring as an additional HIV prevention choice for cisgender women.^5^ The HIV Prevention Trial Network 083 and 084 studies found long-acting injectable cabotegravir superior to oral PrEP at preventing HIV acquisition in cisgender men who have sex with men and transgender women^6^ and cisgender adult women.^7^ These new PrEP products, once more widely available, might further support efforts to decrease global HIV incidence.

**Research in context**

**Evidence before this study**

We searched PubMed for research articles published up to May 19, 2021, with no language restrictions, using the following two sets of search terms: first, “HIV” AND (“pre-exposure prophylaxis” OR “PrEP”) AND “guidelines”, and, second, “HIV” AND (“pre-exposure prophylaxis” OR “PrEP”) AND (“forecast” OR “projection” OR “projecting”). In an online survey on national PrEP guidelines in the European region in 2019 (Moseholm et al, 2020), respondents from 23 countries reported that national PrEP guidelines existed in their countries. Similarly, an online survey in central and eastern Europe (Balayan et al, 2021) found that 40·7% of respondents reported that PrEP was recommended in national guidelines (without specifying countries). A rapid review of PrEP guideline adoption in nine countries in the Asia-Pacific region (Haldar et al, 2021) found that five countries had guidelines in 2020. Hodges-Mameletzis et al (2018) described trends in the adoption of the 2015 WHO recommendation of offering oral PrEP to people at substantial risk into national guidelines globally until 2018. See appendix (p 12) for previous studies cited here. No more recent update on global guideline adoption was identified, and no previous study had analysed routinely collected data on guideline adoption. Global numbers of PrEP users over time are available online through PrEPWatch and UNAIDS. However, these data have limitations or the data cover only a subset of countries. PEPFAR data on PrEP are available online, but these data include only new PrEP initiations in specific PEPFAR-supported countries. No study has compiled global data on trends in both the adoption of PrEP guidelines and numbers of PrEP users. No study was identified that projected global use of oral PrEP based on historical trends observed in early adopting countries.

**Added value of this study**

This study describes global trends in the adoption of the WHO recommendations on PrEP into national guidelines and numbers of PrEP users until the end of 2019. Using a comprehensive and validated dataset compiled through the Global AIDS Monitoring system and WHO, we showed that, by the end of 2019, 120 countries had adopted the WHO PrEP recommendations into national guidelines and that there were about 626 000 PrEP users in 77 countries. We also described PrEP use by priority and vulnerable populations. The observed trends in guideline adoption and PrEP user numbers formed the basis to project future PrEP use, with a projected 2·4–5·3 million PrEP users by the end of 2023 under different growth scenarios.

**Implications of all the available evidence**

Oral PrEP use has been increasing globally. This increase in PrEP users coincides with the widespread adoption of WHO recommendations on PrEP in national guidelines. We projected a four to eight times increase in PrEP use globally by 2023 compared with 2019, but there is considerable scope for expansion of PrEP services globally. In many countries where PrEP services are available, the number of PrEP users remains small relative to numbers of new HIV infections. New PrEP products, such as long-acting injectable cabotegravir and the dapivirine vaginal ring, might further expand PrEP use by offering potential users options other than oral administration. With greater PrEP use, a range of method options, and improved delivery, PrEP programmes might be able to make substantial progress towards the UN Declaration of ending AIDS by 2030.

The 2016 UN General Assembly Political Declaration on Ending AIDS by 2030 included a commitment to ensure 3 million people at high risk of HIV accessed PrEP by 2020.^8^ At that time, PrEP availability was limited to a few, predominately high-income settings, most notably the USA, where nearly 60 000 people (commonly White and affluent men^9^) used PrEP in 2015.^10^ Since 2016, a growing number of countries have adopted WHO recommendations on PrEP into national guidelines, and PrEP services have become increasingly available through pilot projects, implementation studies, and national programmes. As the 2020 global PrEP target ends, and new targets are to be released, it is important to take stock of global PrEP use and access, assess implementation successes and challenges, and consider future scale-up of PrEP. We aimed to describe global trends in the adoption of WHO oral PrEP recommendations into national guidelines, report on patterns in numbers of people who received oral PrEP at least once in a given year, and estimate potential future trajectories of PrEP user numbers. We focus on trends since 2016 because this was when the political declaration was adopted and before which, outside the USA, PrEP was largely limited to small projects. We also limit projections until 2023 because of the large uncertainty associated with longer-term projections.

## Methods

### Data sources

In this global summary and forecasting study, units of analysis were WHO member states (henceforth referred to as countries) across WHO regions (henceforth referred to as regions: 47 countries in the African region, 35 in the region of the Americas, 21 in the Eastern Mediterranean region, 53 in the European region, 11 in the South-East Asia region, and 27 in the Western Pacific region; appendix pp 3–7). The key study indicators were the number of people who had received oral PrEP at least once in a given year (ie, PrEP users; having met local eligibility requirements and been provided PrEP) and the adoption of the WHO recommendations on oral PrEP provision for populations at substantial risk of HIV in national HIV guidelines by countries. Data for these indicators were reported by countries through the UNAIDS and WHO Global AIDS Monitoring (GAM) system and to WHO through regional and country offices. Details on data are provided in the appendix (pp 1–7).

Countries were classified as having adopted WHO PrEP recommendations in national guidelines if they reported “yes” to the GAM question “Has the WHO recommendation on oral PrEP been adopted in your country's national guidelines?” Countries that had adopted WHO PrEP recommendations but were yet to implement these guidelines at time of data collection were also classified as having adopted the recommendations. Countries that reported “no” to this question were classified as pending adoption if they reported plans to adopt PrEP recommendations into guidelines within the next 2 calendar years. Countries reporting no plans, plans with no specified year, or plans for adoption after more than 2 calendar years were classified as no adoption. GAM data for 2016–19 were used to describe trends in adoption of WHO PrEP recommendations. Data reported to WHO were used to fill gaps in GAM data. If a country had reported adoption of WHO PrEP recommendations in a previous year, this information was imputed forward where there were missing data for subsequent years. For GAM data, additional information on the status of implementation of PrEP guidelines was available. This information was used to identify countries that had adopted WHO PrEP recommendations in national guidelines but had not yet started to implement them in 2019.

Numbers of PrEP users by country and region were obtained for 2019 from either GAM or country-specific reporting to WHO as available. Numbers were verified with countries where possible as part of a validation process before finalisation. Where data on PrEP users were available from both the GAM and WHO reporting, higher numbers were used because estimates were more likely to reflect the whole of 2019. Numbers of PrEP users for 2016–18 were taken from a validated WHO dataset that was based on GAM and WHO country reporting data and went through the same verification process with countries as 2019 data. Because national programme data were used, data were unlikely to cover informal PrEP use, although some countries might have included estimates on informal PrEP use in reported data.

For 2019, PrEP user numbers were also obtained by gender and priority population, including adolescent girls and young women (aged 15–24 years), men who have sex with men, sex workers, transgender people, people who inject drugs, and people who live in prison. These data were available for a subset of countries that reported through GAM.

### Forecasting

Numbers of PrEP users were projected globally and by region for each year between 2020 and 2023 in seven different growth scenarios and one COVID-19 disruption scenario. Details on forecasting methods are provided in the appendix (pp 8–10). In short, three countries with at least 3 years of PrEP user data were selected per region as example countries, and PrEP use trends in these countries were used to estimate example PrEP trajectories, representing different HIV epidemics and PrEP programme histories (appendix p 8). In each example country, PrEP user numbers were projected to 2030 by fitting logistic growth curves to observed PrEP user data and an assumed PrEP equilibrium in 2030. This equilibrium was the share of the number of people living with HIV in the example country relative to the global number of people living with HIV multiplied by an assumed global number of 5 million PrEP users. These equilibria do not represent PrEP targets and were only necessary for fitting the example trajectories. Sensitivity analyses were implemented with 3·5 million and 6·5 million global PrEP users as equilibria. In addition to observed growth trajectories, a lower-growth example trajectory was estimated for each example country by reducing PrEP user growth by 25% in each year, so there were six example trajectories in each region, representing various future PrEP use growth scenarios. In some regions, limited available data meant that countries outside these regions were selected as example countries and additional higher-growth example trajectories were estimated to ensure that six different example trajectories were available in each region (appendix p 10). Relative PrEP user growth rates between each year were calculated for each example trajectory. To apply growth rates to all countries in corresponding regions, each country was allocated a step along a PrEP trajectory based on the year they first adopted the WHO PrEP recommendations and reported PrEP users (whichever occurred first), with increasing steps as per the number of continuous years of reported PrEP users. For example, for a country on step 1 of the PrEP trajectory (PrEP use first reported in 2019), growth rates between the first 2 years of example trajectories were applied to the number of PrEP users in that country in 2019 to estimate PrEP users in 2020, followed by growth rates between the second and third years of example trajectories, and so on.

Countries with no PrEP users in 2019 were considered to be on step 0 if the WHO PrEP recommendations were adopted or pending adoption. Since these countries had no reported PrEP use, numbers of PrEP users for 2020, the assumed first year of PrEP introduction, were estimated as PrEP seeds. The PrEP seed was estimated by calculating the mean number of PrEP users reported in the first year of data availability per person living with HIV in a region (2019 data) and multiplying this mean by the number of people with HIV in the seed country. After PrEP seeding, example growth rates were applied for subsequent years of projected growth. Countries without PrEP users in 2019 and where WHO PrEP recommendations were not adopted or pending were excluded from forecasting.

With six example trajectories applied to each country, mean numbers of PrEP users in each year in each country were calculated for the three observed growth example trajectories and the three 25%-reduced trajectories. Means were summed up per region and globally. Additionally, a linear growth scenario was modelled in which increases observed between the past 2 years of data were added every year until 2023 and no new countries were assumed to introduce PrEP services. If a country only had data for 2019, this number was used as the annual increase. Finally, one COVID-19 disruption scenario was modelled in which no growth in 2020 was assumed and PrEP seeding in countries was postponed until 2021. After the disruption, PrEP user growth was modelled with the same trajectories as without disruptions but for only 3 years (2020–23) instead of 4 years in scenarios without disruptions (2019–23).

### Role of the funding source

The funders of the study had no role in the study design, data collection, data analysis, data interpretation, writing of the report, or the decision to submit for publication.

## Results

**Figure 1:**
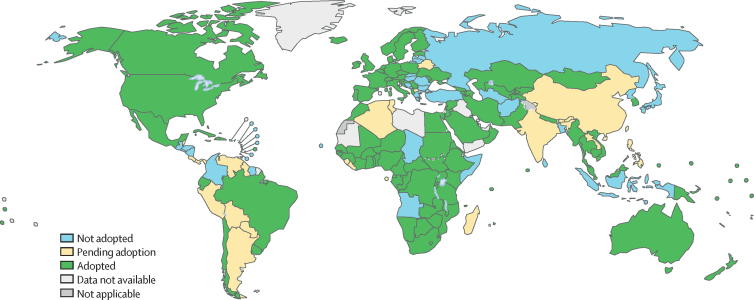
Adoption of the WHO recommendations on oral PrEP into national guidelines globally by 2019 Pending adoption was defined as plans to adopt the recommendation in the next 2 calendar years. Estimates were based on data from the Global AIDS Monitoring system and reporting to WHO. See the appendix (pp 1–7) for details on data. PrEP=pre-exposure prophylaxis.

**Figure 2:**
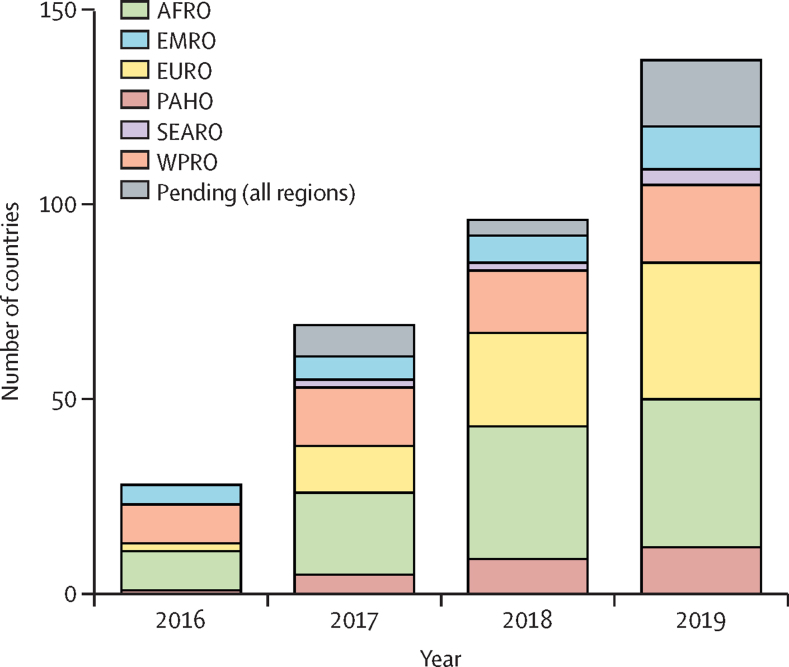
Numbers of countries that have adopted the WHO recommendations on oral PrEP into national guidelines per year by WHO region The figure includes countries that are yet to implement these guidelines. Pending adoption was defined as plans to adopt the recommendation in the next 2 calendar years. Estimates were based on the Global AIDS Monitoring system and reporting to WHO. See the appendix (pp 1–7) for details on data. Regions refer to WHO regional offices. AFRO=African region. EMRO=Eastern Mediterranean region. EURO=European region. PAHO=region of the Americas. PrEP=pre-exposure prophylaxis. SEARO=South-East Asia region. WPRO=Western Pacific region.

For 180 of 194 countries with available data, 120 (67%) had adopted the WHO PrEP recommendations by the end of 2019, with adoption pending in a further 22 (12%) countries in 2020 or 2021 (figure 1). Data on year of adoption were available for 112 countries, and 23 countries adopted the recommendations in 2019, 30 in 2018, 31 in 2017, and 28 in 2016 or earlier (figure 2). In the African region, 38 (81%) of 47 countries had adopted the recommendations, compared with 20 (74%) of 27 in the Western Pacific region, 35 (66%) of 53 in the European region, 11 (52%) of 21 in the Eastern Mediterranean region, four (36%) of 11 in the South-East Asia region, and 12 (34%) of 35 in the region of the Americas. Of 76 countries that reported in GAM that they had adopted the WHO PrEP recommendations into national guidelines, 32 (42%) reported they were yet to implement them.

**Figure 3:**
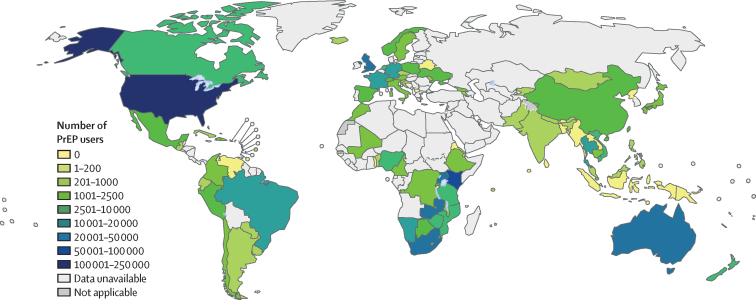
Numbers of people who have received oral PrEP at least once (PrEP users) globally in 2019 Estimates were based on data from the Global AIDS Monitoring system and reporting to WHO. See the appendix (pp 1–7) for details on data. PrEP=pre-exposure prophylaxis.

**Figure 4:**
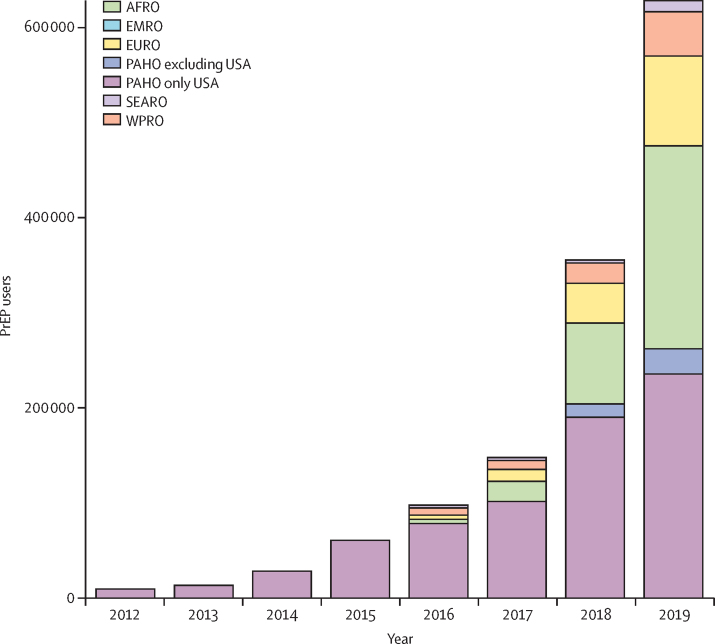
Numbers of people who received oral PrEP at least once (PrEP users) per year by WHO region Data for 2012–15 were added for illustration of longer-term trends and were taken from Sullivan and colleagues,^10^ which were limited to the USA. Estimates for 2016–19 were based on the Global AIDS Monitoring system and reporting to WHO. See the appendix (pp 1–7) for details on data. Regions refer to WHO regional offices. AFRO=African region. EMRO=Eastern Mediterranean region. EURO=European region. PAHO=region of the Americas. PrEP=pre-exposure prophylaxis. SEARO=South-East Asia region. WPRO=Western Pacific region.

Data on PrEP users were available for 96 of 194 countries. In 2019, about 626 000 people received PrEP at least once across 77 countries (figure 3), which represents a 69% increase from the approximately 370 000 PrEP users across 66 countries in 2018 (figure 4). Among global PrEP users in 2019, 260 000 (41·6%) were in the region of the Americas, of whom 234 000 (37·4%) were in the USA, and 213 000 (34·0%) users were in the African region. Information on gender was available for only about 134 000 PrEP users. Among the priority populations, the largest numbers of PrEP users were reported to be men who have sex with men, adolescent girls and young women, and sex workers (table).

**Table:** Oral PrEP users globally by subpopulation in 2019

	**Reporting countries**	**PrEP users in subpopulation/total PrEP users in reporting countries (%)**
**Gender**		
Cisgender male	25	55 564/123 945 (44·8%)
Cisgender female	25	76 444/123 939 (61·7%)
Transgender	26	1871/143 078 (1·3%)
**Priority population**		
AGYW	15	22 413/104 244 (21·5%)
MSM	27	35 990/136 562 (26·4%)
Sex workers	25	24 788/136 297 (18·2%)
PWID	16	870/80 757 (1·1%)
People in prison	15	691/80 229 (0·9%)

PrEP users were defined as people who had received oral PrEP at least once in 2019. Numbers were based on data from the Global AIDS Monitoring system. Reporting countries refers to countries with any data on PrEP users by the specific subpopulations (including reporting of zero PrEP users in a population). These reporting countries vary by population, so the table should be understood as giving an indication of the number and proportion of PrEP users by subpopulation in reporting countries. See the appendix (pp 1–7) for details on data. AGYW=adolescent girls and young women. MSM=men who have sex with men. PrEP=pre-exposure prophylaxis. PWID=people who inject drugs.

**Figure 5:**
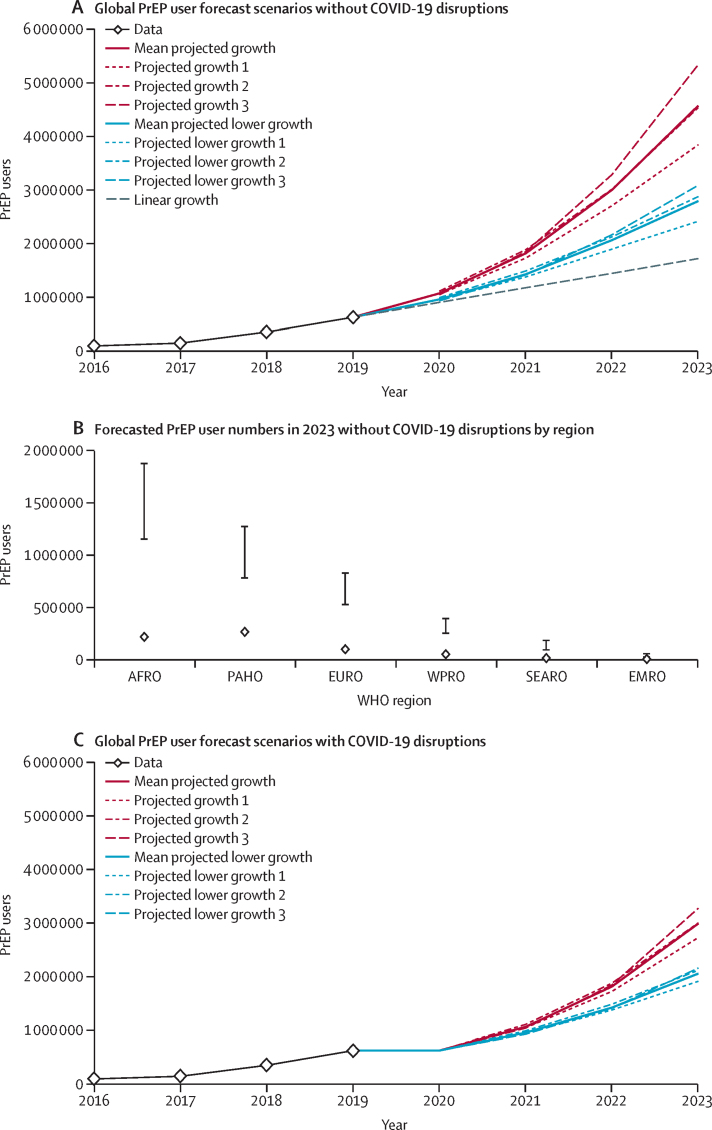
Forecasted global numbers of PrEP users per year until 2023 PrEP users were defined as people who have received oral PrEP at least once. (A) Without COVID-19 disruptions, projections based on three observed example trajectories for each region applied to all countries in the region; lower growth was based on the example trajectories with 25% lower growth at each time step; countries that had adopted the WHO PrEP recommendations or were pending the adoption were assumed to introduce PrEP services in 2020; in the linear growth scenario, the same number of PrEP users was added to each country as observed between the past 2 years of data availability and no country was assumed to introduce PrEP services. (B) Forecasted PrEP users by WHO region without assumed COVID-19 disruptions in 2023; vertical lines represent the average growth scenarios (upper limit) and 25%-reduced growth scenarios (lower limit); diamonds represent 2019 PrEP user numbers. (C) The modelled COVID-19 disruption scenario assumed no growth in 2020 and PrEP seeding in countries was postponed until 2021. After the disruption, growth in PrEP users was modelled with the same trajectories as without COVID-19 disruptions. AFRO=African region. EMRO=Eastern Mediterranean region. EURO=European region. PAHO=region of the Americas. PrEP=pre-exposure prophylaxis. SEARO=South-East Asia region. WPRO=Western Pacific region.

By the end of 2020, between 0·9 and 1·1 million global PrEP users were projected under the example trajectory assumptions and 0·9 million under linear growth assumptions (figure 5A). At the end of 2023, the mean number of people projected to have received PrEP at least once in the year across the three observed growth trajectories is 4·6 million, ranging from 3·8 to 5·3 million across the three example trajectories, and 2·8 million in the 25%-reduced trajectories (range 2·4–3·1 million). 150 000–300 000 PrEP users in 2023 are in 42 countries modelled to introduce PrEP services in 2020. By the end of 2023, most PrEP users are projected to be in the African and Americas regions (figure 5B). The highest relative growth is estimated for the South-East Asia region (90 000–180 000 PrEP users in 2023 *vs* 11 000 in 2019). In sensitivity analyses of different assumptions about future PrEP use in example countries, the mean of the observed growth example trajectories ranged from 3·8 to 5·1 million (appendix p 11). In the COVID-19 disruption scenario, mean numbers of PrEP users by the end of 2023 is 3·0 million in the projected growth scenarios (*vs* 4·6 million in scenarios without disruption; 34% lower), and 2·1 million (*vs* 2·8 million; 26% lower) in the 25%-reduced growth scenarios (figure 5C).

## Discussion

Within 1 year of WHO recommending oral PrEP for anyone at substantial HIV risk, 26 countries had adopted the recommendations into national guidelines, rising to 120 countries in 2019. This widespread adoption of the WHO PrEP recommendations coincides with a global increase in oral PrEP use, and about 626 000 people had received PrEP at least once in 2019. Until 2015, PrEP use was largely limited to the USA,^10^ but sizable numbers of PrEP users can now be found in every region of the world.

More countries had adopted the WHO PrEP recommendations than reported PrEP users in 2019. This finding highlights that, although the adoption of WHO PrEP recommendations into national guidelines is a key step towards PrEP availability in a country, it does not necessarily equate to implementation of PrEP services in all countries or for all populations at an increased risk of HIV. Key facilitators of PrEP implementation include development of operational guidelines and protocols, provider training, supply chains, monitoring and evaluation systems, and demand creation. Cost of PrEP might be a barrier to uptake even in places with national guidelines and might prompt its informal use.^11^

We estimate that there could be 4·6 million PrEP users globally by the end of 2023 if countries follow PrEP use patterns observed in example countries over the past 4 years, with one scenario projecting the number of PrEP users to increase to 5·3 million by the end of 2023. Under the 25% lower-growth scenarios, the projection was 2·8 million PrEP users by the end of 2023. This range of a four to eight times increase in global PrEP users compared with 2019 underscores the uncertainty around future PrEP user growth, but there is substantial scope for expansion of PrEP services globally. 32 countries reported in GAM that they adopted WHO PrEP recommendations into national guidelines but are yet to implement them, and 22 countries reported that they plan to adopt WHO recommendations on PrEP in 2020 or 2021. In 2020, tenofovir disoproxil fumarate with emtricitabine was approved for HIV prevention in China, where more than 130 000 people acquired HIV in 2019.^12^ Other countries where WHO PrEP recommendations are pending adoption are also characterised by large numbers of new HIV infections (eg, 16 000 in the Philippines in 2019^13^), often concentrated among key populations. In many countries where PrEP services are available, the number of PrEP users remains small relative to their HIV epidemics and numbers of new infections, which highlights the often slow transition from demonstration and pilot projects to large-scale programmes. Even in countries with relatively large numbers of PrEP users, such as South Africa and Kenya, there might be substantial scope for further expansion in PrEP use because of the large numbers of new HIV infections. This suggests that, in many countries, large numbers of people remain at increased risk of HIV, so there is strong potential for further growth in global numbers of PrEP users and for population-level impact if sufficient coverage can be achieved.

New PrEP products and formulations, including long-acting cabotegravir and the dapivirine vaginal ring, might further expand PrEP use by offering potential users additional options to daily or event-driven oral administration, as occurred when contraceptive options increased.^14^ Global investments in PrEP continue to support the introduction and use of these methods. These investments include large financial commitments by the US President's Emergency Plan for AIDS Relief (PEPFAR), which aimed for 340 000 new PrEP users in priority countries in 2020 alone, and other international funding organisations such as The Global Fund to Fight AIDS, Tuberculosis and Malaria, as well as increasing funding for PrEP from private organisations^15^ and by some national governments such as in Thailand. Therefore, considerable increases in global PrEP users over the next few years could be achievable.

Our modelled COVID-19 disruption scenario suggests that, if the numbers of global PrEP users stay the same in 2020 as they were in 2019, it could lead to substantial cumulative reductions of PrEP users in 2023 compared with scenarios without the disruption, even if growth rates were assumed to return to previous levels in 2021. This is because such a reduction in PrEP users would only be offset by increasing annual growth rates above those applied in our modelled trajectories. The actual impact of COVID-19 on PrEP service provision for 2020 is unclear. Although disruptions in PrEP use^16^, ^17^, ^18^ and PrEP service delivery^19^, ^20^ occurred in many places, there were more than 300 000 new PrEP initiations across PEPFAR-supported programmes in 2020, suggesting that PrEP user numbers continued to grow globally in 2020. Nevertheless, results of modelled COVID-19 disruption underscore the importance of adapting programmes to address barriers to access and continued engagement in PrEP services. Such adaptations, some of which were widely adopted during COVID-19 restrictions, include multimonth dispensing of PrEP, the use of telemedicine,^19^, ^21^ streamlined service delivery, HIV self-testing, and other community-based modes of PrEP delivery. These community-based models of PrEP delivery, including use of lay providers,^22^ have the potential to improve uptake of and adherence to PrEP,^23^, ^24^ which were gaps in the PrEP cascade before the effects of COVID-19.^23^

In the limited subset of countries that reported PrEP use among priority and vulnerable populations, the largest numbers of PrEP users were reported to be men who have sex with men, adolescent girls and young women, and sex workers. Few people who inject drugs and people who live in prison were reported to have received PrEP, supporting findings of a systematic review that PrEP use among people who inject drugs is low.^25^ In mathematical modelling studies, focusing on priority populations with typically higher HIV incidence rates than the general population is most cost-effective,^26^ and these populations are often prioritised in PrEP policies. Moreover, observational studies in high-income settings suggest that provision of PrEP to key populations, and achieving high coverage, has contributed to population-level reduction in HIV incidence.^27^, ^28^ The inclusion of PrEP services for adolescent girls and young women, primarily in the African region, has resulted in a large increase in young women having received PrEP. This increasing use of PrEP has potential to decrease HIV incidence, particularly in more generalised epidemics,^29^ although PrEP continuation has been a challenge.^23^

Although we made every effort to obtain the most up-to-date PrEP user data and to ensure that these data had been verified, information was missing for some countries. Some countries reported PrEP use in previous years but not in subsequent years. In some places, this change in reporting might have been due to pilot or demonstration programmes that ended. These missing data might have led to underestimation of numbers of PrEP users. Oral PrEP might be available through informal channels (eg, online) and private health-care systems in countries without PrEP policy or where PrEP users in the private system are not reported. Our data are unlikely to capture use of PrEP obtained through such informal channels or behaviours such as pill sharing or use of post-exposure prophylaxis as PrEP, which might also have led to an underestimation of the number of PrEP users. By contrast, some countries might report cumulative PrEP users as current PrEP users because better information is unavailable. Despite these limitations, efforts to verify data and use of multiple data sources suggest that global numbers of PrEP users are higher than reported elsewhere.^30^ A limitation of our measure of PrEP users was that it might not reflect need for PrEP, which would, ideally, be based on size estimates for key and vulnerable populations and consider proportions of these populations at substantial HIV risk; however, these data vary a lot in quality and are not widely available. Similarly, data distinguishing between new and continuing PrEP users and for length of time on PrEP were sparse, so we were unable to evaluate patterns of PrEP use. Therefore, our results on numbers of people who received PrEP at least once in a year need to be interpreted with caution because they might not represent effective use of PrEP and translate to population-level impact. Finally, data on gender and priority populations were limited to a subset of countries. These data might not be representative of the global or regional situation (eg, the share of cisgender males among all PrEP users was probably higher than suggested by the data), and it was not possible to evaluate differences in PrEP user characteristics by region.

PrEP projections were dependent on historical PrEP data. Therefore, countries might have been misclassified as being early on the PrEP trajectory because of missing data and modelled to experience higher growth than they would have with more accurate information. By contrast, future PrEP growth could not be estimated for some countries, predominately countries with small population sizes, because data on people living with HIV were unavailable, so no PrEP seed could be estimated. The PrEP seeding approach also assumes that 42 countries will introduce PrEP services in 2020 on the basis of their reported intentions. This number is larger than the number of countries that started reporting any PrEP use in 2019 (ten countries) or that adopted the WHO PrEP recommendations in 2019 (23 countries). However, even if introduction timelines shift, the effect of these PrEP introductions on global numbers was limited because they represented a small proportion of total projected PrEP users. Moreover, PrEP seeds were estimated based on numbers of people living with HIV in a country rather than new HIV infections. Although HIV incidence might be a better measure of PrEP need, global data on numbers of people with HIV were more complete and, given the small numbers of projected PrEP users in PrEP seeding countries, the effect of using a different metric was probably negligible. Finally, our PrEP forecasting depended on a selection of example countries for which trajectories were estimated. Long-term projections in these example countries might be unrealistic. However, relative growth in example trajectories in early years after the introduction of PrEP was driven by observed data. Because most countries were early on their PrEP trajectories and PrEP users were forecasted only until 2023, the effect of the long-term projections in the example countries on global PrEP user forecasts was limited. The selection of the example countries was driven by availability of PrEP use data from multiple years in these countries. Although the observed growth in these example countries might not be representative for all countries in a region (particularly for countries that have only recently or not yet introduced PrEP services), six different example trajectories were estimated for each country, representing a range of possible future PrEP use growth scenarios. The results of these different scenarios, as well as of those with different assumptions of future PrEP use in example countries, highlight the uncertainty around growth of PrEP use.

To conclude, numbers of PrEP users have increased substantially and become more global since WHO recommended oral PrEP for people at substantial risk of HIV in 2015. Although the target of 3 million PrEP users by 2020 will be missed, PrEP use is likely to continue to rise at an accelerated pace with continuing adoption and implementation of PrEP policies by countries. The integration of PrEP services, including long-acting methods, with other health services, such as sexual and reproductive health services,^31^, ^32^ might improve uptake and adherence and can further increase the efficiency of service delivery. With greater implementation of PrEP services, increasing awareness, demand, and uptake, a range of method options, and simplified delivery, public health programmes have better opportunities to reach individuals who could benefit from PrEP, prevent new HIV infections, and make substantial progress towards the UN declaration of ending AIDS by 2030.

## Data sharing

GAM data (numbers of PrEP users and WHO PrEP recommendations adoption) used for this study are available online. Additional data obtained through WHO regional and country offices are available on request to the corresponding author.

## Declaration of interests

We declare no competing interests.
