# Supplementary material for: Adoption of guidelines on and use of oral pre-exposure prophylaxis: a global summary and forecasting study
Source: Lancet HIV. 2021 Jul 12;8(8):e502–10. doi: 10.1016/S2352-3018(21)00127-2 (PMC8332196; doi:10.1016/S2352-3018(21)00127-2)
Supplement: Supplementary appendix [file mmc1.pdf]

THE LANCET
HIV

Supplementary appendix

This appendix formed part of the original submission and has been peer reviewed. We post it as supplied by the authors.

Supplement to: Schaefer R, Schmidt H-MA, Ravasi G, et al. Adoption of guidelines on and use of oral pre-exposure prophylaxis: a global summary and forecasting study. *Lancet HIV* 2021; published online July 12. http://dx.doi.org/10.1016/S2352-3018(21)00127-2.

Global adoption of guidelines on and use of oral pre-exposure prophylaxis (PrEP): Current situation and future projections

Schaefer et al.

Supplementary material

Details on data

The key indicators for this study were the number of people who have received oral PrEP at least once in a given year ('PrEP users') (having met local eligibility requirements and been provided PrEP) and the adoption the WHO recommendations on oral PrEP provision for populations at substantial risk of HIV acquisition in national HIV guidelines by countries ('adoption of the WHO PrEP recommendations'). Data on these indicators were obtained through the Global AIDS Monitoring (GAM). Countries report on these indicators in the GAM for the previous year in the first half of a given year. Details on the GAM can be found elsewhere [1]. Table S1 below lists the survey items used in the GAM to collect information. To validate GAM data and fill data gaps, WHO reached out to regional offices to collect information with the same survey instruments. Table S2 lists the data sources used for each indicator for each WHO member state by WHO region for 2019. If a country was known to have adopted the WHO PrEP recommendations in a previous year but reported missing data in a subsequent year, this was imputed. For numbers of PrEP users for 2016-2018, a validated WHO data set was used. Table S2 also lists the allocated step of on the 'PrEP trajectory' used for the PrEP user forecasting (see below).

Table S1: Survey items used to gather information on numbers of PrEP users and WHO PrEP recommendations adoption.

Survey item	Response possibilities
Numbers of PrEP users	
Number of people who received oral PrEP at least once during the reporting period	Disaggregation:<br>• People who received PrEP for the first time in their lives.<br>• Gender (male, female or transgender).<br>• Age (<15, 15+, 15-19, 20-24-25-49 and 50+ years)<br>• Key population: (meh who have sex with men, sex workers, people who inject drugs, and transgender people and prisoners)<br>• Cities and other administrative areas of importance
Adoption of the WHO PrEP recommendations	
Has the WHO recommendation on oral PrEP been adopted in your country's national guidelines?	Yes, PrEP guidelines have been developed and are being implemented<br>Yes, PrEP guidelines have been developed but are not yet being implemented<br>No, guidelines have not been developed
If the WHO recommendation on oral PrEP has not yet been adopted in the national guidelines, is there a plan to adopt a PrEP recommendation in the future?	Yes<br>No
If yes, please indicate the year when adoption of the PrEP recommendations is planned:	No planned year<br>Year 1 (depending on year of data collection)<br>Year 2 (depending on year of data collection)<br>Year 3 (depending on year of data collection)<br>Year 4 (depending on year of data collection)<br>Other (please specify)

Table S2: Data sources for numbers of PrEP users and adoption of the WHO recommendations on PrEP in 2019, and the allocated PrEP trajectory step for PrEP user forecasting (see below), by WHO region and country.

**Country**	**Region**	**PrEP user data source**	**WHO PrEP recommendation adoption data source**	**PrEP trajectory step (forecasting)** [a]
		WHO Regional Office for the African Region: AFRO		
Algeria	AFRO	No data	No data	NA [b]
Angola	AFRO	No data	GAM	NA [b]
Benin	AFRO	WHO	GAM	3
Botswana	AFRO	GAM	GAM	4
Burkina Faso	AFRO	No data	GAM	0
Burundi	AFRO	No data	GAM	0
Cabo Verde	AFRO	No data	WHO	NA [b]
Cameroon	AFRO	GAM	GAM	2
Central African Republic	AFRO	No data	GAM	0
Chad	AFRO	No data	WHO	NA [b]
Comoros	AFRO	No data	GAM	0
Congo	AFRO	No data	WHO	0
Côte d'Ivoire	AFRO	No data	GAM	0
Democratic Republic of the Congo	AFRO	WHO	WHO	3
Equatorial Guinea	AFRO	No data	WHO	0
Eritrea	AFRO	WHO	WHO	0
Eswatini	AFRO	GAM	WHO	4
Ethiopia	AFRO	GAM	GAM	2
Gabon	AFRO	No data	GAM	0
Gambia	AFRO	GAM	WHO	1
Ghana	AFRO	No data	GAM	0
Guinea	AFRO	No data	GAM	0
Guinea-Bissau	AFRO	No data	GAN	0
Kenya	AFRO	WHO	GAM	6
Lesotho	AFRO	GAM	GAM	6
Liberia	AFRO	No data	WHO	0
Madagascar	AFRO	No data	WHO	0
Malawi	AFRO	GAM	GAM	2
Mali	AFRO	WHO	WHO	2
Mauritania	AFRO	No data	No data	NA [b]
Mauritius	AFRO	GAM	GAM	3
Mozambique	AFRO	GAM	WHO	3
Namibia	AFRO	WHO	GAM	4
Niger	AFRO	No data	GAM	0
Nigeria	AFRO	GAM	GAM	4
Rwanda	AFRO	WHO	WHO	2
Sao Tome and Principe	AFRO	No data	WHO	NA [b]
Senegal	AFRO	No data	GAM	0
Seychelles	AFRO	GAM	GAM	4
Sierra Leone	AFRO	No data	GAM	0
South Africa	AFRO	GAM	GAM	6
South Sudan	AFRO	No data	GAM	0
Togo	AFRO	WHO	GAM	0
Uganda	AFRO	GAM	WHO	6
United Republic of Tanzania	AFRO	WHO	GAM	4
Zambia	AFRO	WHO	GAM	6
Zimbabwe	AFRO	WHO	GAM	4

[a] Each country was allocated a step on a 'PrEP trajectory' to forecast future PrEP user numbers. See below for details.
[b] The country was not included in the PrEP forecasting either because it was not considered to be on a 'PrEP trajectory' or because no 'PrEP seed' could be estimated due to lack of data on numbers of people living with HIV. See below for details.

Table S2 cont.

Country	Region	PrEP user data source	WHO PrEP recommendation adoption data source	PrEP trajectory step (forecasting) [a]
		WHO Regional Office for the Eastern Mediterranean Region: EMRO		
Afghanistan	EMRO	No data	WHO	NA [b]
Bahrain	EMRO	No data	No data	NA [b]
Djibouti	EMRO	No data	No data	NA [b]
Egypt	EMRO	No data	GAM	0
Iran (Islamic Republic of)	EMRO	No data	GAM	0
Iraq	EMRO	No data	No data	NA [b]
Jordan	EMRO	No data	GAM	NA [b]
Kuwait	EMRO	No data	GAM	NA [b]
Lebanon	EMRO	WHO	WHO	1
Libya	EMRO	No data	No data	NA [b]
Morocco	EMRO	GAM	GAM	3
Oman	EMRO	No data	GAM	0
Pakistan	EMRO	WHO	WHO	1
Qatar	EMRO	No data	No data	NA [b]
Saudi Arabia	EMRO	No data	GAM	NA [b]
Somalia	EMRO	No data	GAM	NA [b]
Sudan	EMRO	No data	GAM	0
Syrian Arab Republic	EMRO	No data	GAM	0
Tunisia	EMRO	No data	WHO	0
United Arab Emirates	EMRO	No data	No data	NA [b]
Yemen	EMRO	No data	No data	NA [b]

[a] Each country was allocated a step on a ‘PrEP trajectory’ to forecast future PrEP user numbers. See below for details.

[b] The country was not included in the PrEP forecasting either because it was not considered to be on a ‘PrEP trajectory’ or because no ‘PrEP seed’ could be estimated due to lack of data on numbers of people living with HIV. See below for details.

Table S2 cont.

Country	Region	PrEP user data source	WHO PrEP recommendation adoption data source	PrEP trajectory step (forecasting) [a]
		WHO Regional Office for the European Region: EURO		
Albania	EURO	No data	GAM	0
Andorra	EURO	No data	No data	NA [b]
Armenia	EURO	GAM	GAM	3
Austria	EURO	WHO	WHO	3
Azerbaijan	EURO	No data	GAM	0
Belarus	EURO	WHO	GAM	0
Belgium	EURO	No data	WHO	NA [b]
Bosnia and Herzegovina	EURO	No data	WHO [c]	NA [b]
Bulgaria	EURO	No data	WHO [c]	NA [b]
Croatia	EURO	WHO	WHO	2
Cyprus	EURO	WHO	WHO [c]	2
Czech Republic	EURO	WHO	WHO	2
Denmark	EURO	No data	WHO	NA [b]
Estonia	EURO	No data	WHO	NA [b]
Finland	EURO	No data	WHO	NA [b]
France	EURO	WHO	WHO	4
Georgia	EURO	GAM	GAM	3
Germany	EURO	WHO [d]	WHO [c]	4
Greece	EURO	No data	WHO [c]	NA [b]
Hungary	EURO	No data	WHO [c]	NA [b]
Iceland	EURO	WHO	WHO	2
Ireland	EURO	No data	WHO	0
Israel	EURO	No data	WHO	NA [b]
Italy	EURO	WHO	WHO [c]	2
Kazakhstan	EURO	No data	GAM	0
Kyrgyzstan	EURO	GAM	GAM	3
Latvia	EURO	No data	WHO [c]	NA [b]
Lithuania	EURO	No data	WHO [c]	NA [b]
Luxembourg	EURO	WHO	WHO [c]	3
Malta	EURO	No data	WHO	NA [b]
Monaco	EURO	No data	GAM	NA [b]
Montenegro	EURO	No data	GAM	NA [b]
Netherlands	EURO	WHO	WHO	3
North Macedonia	EURO	WHO	WHO	NA [b]
Norway	EURO	WHO	WHO	5
Poland	EURO	WHO	WHO	2
Portugal	EURO	No data	WHO	NA [b]
Republic of Moldova	EURO	GAM	GAM	2
Romania	EURO	No data	WHO [c]	NA [b]
Russian Federation	EURO	No data	WHO [c]	NA [b]
San Marino	EURO	No data	No data	NA [b]
Serbia	EURO	No data	GAM	0
Slovakia	EURO	No data	WHO [c]	NA [b]
Slovenia	EURO	WHO	WHO [c]	1
Spain	EURO	WHO	WHO	2
Sweden	EURO	WHO	WHO	3
Switzerland	EURO	WHO	WHO	4
Tajikistan	EURO	No data	GAM	0
Turkey	EURO	No data	GAM	NA [b]
Turkmenistan	EURO	No data	WHO	NA [b]
Ukraine	EURO	GAM	WHO	4
United Kingdom of Great Britain and Northern Ireland	EURO	WHO	WHO	4
Uzbekistan	EURO	No data	GAM	0

[a] Each country was allocated a step on a 'PrEP trajectory' to forecast future PrEP user numbers. See below for details.
[b] The country was not included in the PrEP forecasting either because it was not considered to be on a 'PrEP trajectory' or because no 'PrEP seed' could be estimated due to lack of data on numbers of people living with HIV. See below for details.
[c] Data were obtained by the WHO Regional Office for Europe through the European Centre for Disease Prevention and Control (ECDC) as published here: https://www.ecdc.europa.eu/sites/default/files/documents/HIV-pre-exposure-prophylaxis-evidence-2019_0.pdf
[d] Data for PrEP users in Germany were based on a publication by the Robert Koch Institute, Germany: https://www.researchsquare.com/article/rs-156075/v2

Table S2 cont.

Country	Region	PrEP user data source	WHO PrEP recommendation adoption data source	PrEP trajectory step (forecasting) [a]
		Pan-American Health Organization (Americas Region): PAHO		
Antigua and Barbuda	PAHO	No data	GAM	NA [b]
Argentina	PAHO	WHO	WHO	1
Bahamas	PAHO	WHO	WHO	4
Barbados	PAHO	GAM	WHO	3
Belize	PAHO	No data	WHO	NA [b]
Bolivia (Plurinational State of)	PAHO	No data	GAM	0
Brazil	PAHO	GAM	GAM	3
Canada	PAHO	WHO	GAM	3
Chile	PAHO	GAM	GAM	1
Colombia	PAHO	GAM	WHO	1
Costa Rica	PAHO	No data	WHO	0
Cuba	PAHO	GAM	WHO	2
Dominica	PAHO	No data	WHO	NA [b]
Dominican Republic	PAHO	GAM	WHO	2
Ecuador	PAHO	GAM	GAM	2
El Salvador	PAHO	No data	GAM	NA [b]
Grenada	PAHO	WHO	WHO	NA [b]
Guatemala	PAHO	GAM	GAM	3
Guyana	PAHO	No data	WHO	0
Haiti	PAHO	GAM	WHO	2
Honduras	PAHO	No data	WHO	NA [b]
Jamaica	PAHO	No data	WHO	0
Mexico	PAHO	WHO	GAM	2
Nicaragua	PAHO	No data	GAM	NA [b]
Panama	PAHO	No data	WHO	NA [b]
Paraguay	PAHO	WHO	WHO	1
Peru	PAHO	GAM	WHO	2
Saint Kitts and Nevis	PAHO	No data	WHO	NA [b]
Saint Lucia	PAHO	No data	WHO	NA [b]
Saint Vincent and the Grenadines	PAHO	No data	WHO	NA [b]
Suriname	PAHO	No data	WHO	NA [b]
Trinidad and Tobago	PAHO	No data	WHO	NA [b]
United States of America	PAHO	WHO [e]	WHO	8
Uruguay	PAHO	GAM	WHO	4
Venezuela (Bolivarian Republic of)	PAHO	WHO	WHO	0

[a] Each country was allocated a step on a 'PrEP trajectory' to forecast future PrEP user numbers. See below for details.

[b] The country was not included in the PrEP forecasting either because it was not considered to be on a 'PrEP trajectory' or because no 'PrEP seed' could be estimated due to lack of data on numbers of people living with HIV. See below for details.

[e] Data for PrEP users in the US were based on information provided by Gilead Sciences, Inc., in the quarterly investors' earnings call for the fourth quarter of 2019.

Table S2 cont.

Country	Region	PrEP user data source	WHO PrEP recommendation adoption data source	PrEP trajectory step (forecasting) [a]
		WHO Regional Office for the South-East Asia Region: SEARO		
Bangladesh	SEARO	WHO	WHO	NA [b]
Bhutan	SEARO	WHO	WHO	NA [b]
Democratic People's Republic of Korea	SEARO	WHO	WHO	NA [b]
India	SEARO	WHO	WHO	4
Indonesia	SEARO	WHO	GAM	NA [b]
Maldives	SEARO	WHO	WHO	NA [b]
Myanmar	SEARO	WHO	GAM	0
Nepal	SEARO	WHO	WHO	2
Sri Lanka	SEARO	WHO	WHO	NA [b]
Thailand	SEARO	WHO	GAM	3
Timor-Leste	SEARO	WHO	WHO	NA [b]
		WHO Regional Office for the Western Pacific Region: WPRO		
Australia	WPRO	WHO	WHO	4
Brunei Darussalam	WPRO	GAM	No data	NA [b]
Cambodia	WPRO	GAM	WHO	1
China	WPRO	WHO	WHO	2
Cook Islands	WPRO	No data	No data	NA [b]
Fiji	WPRO	WHO	WHO	0
Japan	WPRO	WHO	WHO	2
Kiribati	WPRO	No data	WHO	NA [b]
Lao People's Democratic Republic	WPRO	WHO	WHO	0
Malaysia	WPRO	WHO	WHO	3
Marshall Islands	WPRO	No data	WHO	NA [b]
Micronesia (Federated States of)	WPRO	No data	WHO	NA [b]
Mongolia	WPRO	GAM	WHO	4
Nauru	WPRO	No data	WHO	NA [b]
New Zealand	WPRO	WHO	GAM	3
Niue	WPRO	No data	No data	NA [b]
Palau	WPRO	No data	WHO	NA [b]
Papua New Guinea	WPRO	WHO	WHO	0
Philippines	WPRO	WHO	GAM	2
Republic of Korea	WPRO	No data	WHO	NA [b]
Samoa	WPRO	No data	WHO	NA [b]
Singapore	WPRO	WHO	WHO	1
Solomon Islands	WPRO	No data	WHO	NA [b]
Tonga	WPRO	No data	WHO	NA [b]
Tuvalu	WPRO	No data	WHO	NA [b]
Vanuatu	WPRO	No data	WHO	NA [b]
Viet Nam	WPRO	GAM	WHO	3

[a] Each country was allocated a step on a 'PrEP trajectory' to forecast future PrEP user numbers. See below for details.

[b] The country was not included in the PrEP forecasting either because it was not considered to be on a 'PrEP trajectory' or because no 'PrEP seed' could be estimated due to lack of data on numbers of people living with HIV. See below for details.

Details on PrEP user forecasting

PrEP trajectories

In order to project numbers of PrEP users to 2023, each country was allocated a 'step' along a 'PrEP trajectory' based on the year they first adopted WHO PrEP recommendations and reported PrEP users (whichever came first), with increasing steps as per the number of continuous years of reported PrEP users up to and including 2019. For instance, a country reporting PrEP use first in 2019 was on 'step 1', while a country reporting PrEP use in both 2018 and 2019 was on 'step 2'. Countries that did not report any PrEP users in 2019 (either zero use or missing data) were considered on 'step 0 'if the WHO PrEP recommendations were adopted or 'pending 'adoption (regardless of any PrEP users reported in years before 2019). Countries that did not report any PrEP users in 2019 and where WHO PrEP recommendations were not adopted or pending were not considered to be on a PrEP trajectory (regardless of any PrEP users reported before 2019). These countries were excluded from the forecasting. Table S2 lists the PrEP trajectory steps for each country and countries that were excluded from the forecasting.

Example PrEP trajectories

To forecast PrEP user trends in countries, two to three example countries with at least three years' of current PrEP user data available were selected per region as 'example PrEP trajectories'. These represented different HIV epidemics and PrEP programme histories (Table S3). The selection of example countries was driven by availability of historic PrEP data in countries as a greater number of data points improves the estimation of the example PrEP trajectories. Where possible, example countries were those with four years of PrEP user data (from 2016-2019). Countries with small numbers of PrEP users in 2019 (under 100) were not considered as candidates for example countries as PrEP user numbers were likely to fluctuate from year to year. Countries with a considerable decrease in reported PrEP users between any two consecutive years (10% or more) were not considered example country candidates as this may have suggested the ending a PrEP study or pilot project without subsequent implementation of a national programme. Example countries were also selected to represent different HIV epidemics and PrEP programme histories as well as different sub-regions within regions. Where there were insufficient numbers of countries with four years of current PrEP user data, countries with three years (2017-2019) were considered as candidates for example countries. There were only two instances where there were more than three candidate countries per region as per these selection criteria. In the African region, Kenya, Nigeria, South Africa, and Zimbabwe were identified as possible example countries. Nigeria and Kenya were selected to represent the western and eastern African sub-regions, respectively. South Africa was selected over Zimbabwe due to the fact that the observed trajectory in Zimbabwe was very similar to the trajectory in Kenya. In the European region, France and the UK were both possible candidates to represent the western Europe (high-income) sub-region; however, at the time of the implementation of the forecasting methodology, the data from the UK had not yet been received in full, so it France was selected.

In the regions where there were insufficient numbers of example country candidates, example countries from other regions were selected based on similarity in HIV epidemics, PrEP programme histories, and geographic proximity. For the Eastern Mediterranean region, where there were no data that met our criteria, Nigeria and Georgia were selected due to their similarly concentrated HIV epidemics and geographic proximity. Other example countries in the African region (South Africa and Kenya) and European region (France and Ukraine) were considered less suitable example candidates for this region. Similarly, for the South-East Asia region, Viet Nam was selected as a suitable candidate due to its similar epidemic profile and geographic proximity.

Given historic trends in PrEP user growth that indicate exponential growth, logistic growth curves (initial slow growth that turns into exponential growth and then increasing flattening of the curve) were fitted to the PrEP user data and an assumed 'PrEP equilibrium' in each example country by minimising the squared error between data and fitted curve. The PrEP user equilibrium represents the number of PrEP users will be reached by 2030. PrEP equilibria were calculated as a share of an assumed global level of five million PrEP users in 2030 depending on the number of people living with HIV (PLHIV) in the country. For instance, 7.5 million of the global 38 million PLHIV in 2019 (19.7%) were living in South Africa [2], so the PrEP equilibrium was 986,842 PrEP users in 2030 (which is 19.7% of all those assumed to be on PrEP in 2030). Numbers of PLHIV instead of HIV incidence was used due to better global data availability. PrEP equilibria do not represent PrEP targets. The PrEP equilibrium in 2030 were necessary for example trajectory curve fitting but effects on forecasting of PrEP user trends in countries were limited as this was restricted to 2023 (see results of a sensitivity analysis below). Logistic growth curves were fitted as

$$PrEP_t = \frac{PrEP_{max}}{1 + e^{-a(t \times \mathrm{t}_{max})}}$$

Where $PrEP_t$ is the number of PrEP users at time t, $PrEP_{max}$ is the maximum number of PrEP users that will be reached by 2030 (the pre-specified PrEP equilibrium), t_{max} is the time point of maximum growth (after which the growth curve starts to flatten), and a is growth parameter. The squared difference between the fitted curve and the observed difference was the error for the curve fitting. The parameters a and t_{max} were optimised to minimise the error with a Generalized Reduced Gradient (GRG) Nonlinear method using Microsoft Excel Solver. Since it was considered important that the growth rates early in the fitted example PrEP trajectories appropriately reflect the PrEP number growth observed in these countries (given that these early growth rates was most important for global projections until 2023), the first value of the fitted curve was constrained to be the same as the first observed number of PrEP users in the example country. This ensured that the fitted curve accurately reflects the growth observed in the first few years after PrEP introduction in the example countries.

After fitting the logistic growth curve to each example country, the growth rate between each two successive years was calculated for the example trajectory. In addition to the 'observed growth trajectory' in each example country, a lower growth example trajectory was estimated by multiplying the growth rate between each two years by 0.75. In the Eastern Mediterranean and South-East Asia region, only two countries had sufficient PrEP user data to calculate example trajectories in the Eastern Mediterranean and South East-Asia regions, so there would only be four example trajectories (two observed and two reduced growth trajectories). The example countries in these regions had relatively low growth rates compared to some other example countries in other regions. For instance, in Nigeria (an example country for the Eastern Mediterranean region), the average growth per year in the first three years after PrEP introduction (based on the available data) was 117%, compared to 287% in South Africa or 188% in Kenya (the two other example countries for the African region). This is similarly true for Thailand (116%) (in the South-East Asia region). To model the possibility that countries in these two regions experience higher growth than in these example countries, an additional 'higher growth trajectory' was created by multiplying the observed growth rate by 1.25 at each time point. This means that there were a total of six trajectories per region. The impact of including this additional higher growth rate trajectory on global estimates of PrEP users was relatively limited as only about 1.8% of PrEP users were projected to reside in these two regions in 2023 (see main article).

Applying example PrEP trajectories
The relative PrEP user growth rates between each year was were applied to all countries in the corresponding region, depending on the country's step on the PrEP trajectory. For instance, for a country on 'step 1' of the PrEP trajectory, growth rates between the first two years of the example trajectories were applied to the number of PrEP users in that country in 2019 to estimate PrEP users in 2020, followed by growth rates between the second and third years of the example trajectories, and so on.

All PrEP trajectories in a region were also applied to the example countries. The PrEP equilibria for these example countries only constrained one of the fitted trajectory for these countries (as well as the 25% lower trajectory); the other four trajectories in the region where applied to the example country without being constrained by the PrEP equilibrium. The PrEP equilibrium was also only relevant for the fitting of the example trajectories in the example countries. Projections in other countries were not constrained by the equilibrium.

PrEP seeding
For countries without PrEP user data for 2019 but that were on step 0 of the PrEP trajectory, a 'PrEP seed was estimated for 2020 to which example growth rates were applied. PrEP seeds were estimated as the mean number of PrEP users reported in the first year of data availability per PLHIV in a region multiplied by the number of PLHIV in a country (using 2019 data for PLHIV). After PrEP seeding, example growth rates were applied for subsequent years of projected growth.

Calculating future PrEP user numbers in different scenarios
With the six example trajectories applied, the average number of PrEP users in each year in each country was calculated for the three observed growth rate example trajectories and the three 25%-reduced trajectories. These means were summed up per region and globally. Additionally, a linear growth scenario was modelled in which the increase observed between the last two years of data was added every year until 2023 and no new countries were assumed to have PrEP services. Finally, one COVID-19 disruption scenario was modelled in which no growth in 2020 was assumed and PrEP seeding in countries was postponed until 2021. After the 2020 disruption, PrEP user growth was modelled with the same trajectories as in scenarios without disruptions.

Table S3: PrEP example trajectories and average PrEP seeds per WHO region for PrEP user forecasting.

Region	Regional PrEP seed per 100,000 PLHIV [a]	Example PrEP trajectories	
		**Example country**	**Assumed PrEP equilibrium in 2030 for example country [b]**
AFRO	176.8	South Africa	986842
		Kenya	197368
		Nigeria	236842
EMRO	533.3 [c]	Nigeria [d]	236842
		Georgia [d]	1197
EURO	1785.7	France	50000 [e]
		Ukraine	32894
		Georgia	1197
PAHO	333.4	Guatemala	4737
		Brazil	121053
		USA	315789 [f]
SEARO	139.3	Thailand [g]	61842
		Viet Nam [g]	30263
WPRO	5376.6	Australia	76316 [h]
		Viet Nam	30263
		Thailand [i]	61842

Regions refer to the WHO regions: AFRO: African Regional Office; EMRO: Eastern Mediterranean Regional Office; EURO: European Regional Office; PAHO: Pan-American Health Organization (Americas region); SEARO: South-East Asia Regional Office; WPRO: Western Pacific Regional Office.

[a] The PrEP seed was calculated as the average number of PrEP users in the year of first data availability across WHO member states in a region per people living with HIV (PLHIV).

[b] PrEP targets for each example PrEP trajectory was based on the 'PrEP need', calculated as the number of PLHIV in the country as a share of all PLHIV multiplied by an assumed global PrEP target of five million PrEP users by 2030.

[c] Due to limited data availability in the EMRO region, the PrEP seed was based on the two countries with available data (Morocco and Pakistan) and the data from the example countries Nigeria and Georgia.

[d] No example country with at least three years of PrEP user data was available for the EMRO region. Nigeria (AFRO) and Georgia (EURO) were selected as example countries to represent concentrated HIV epidemics. To generate six example trajectories, PrEP user growth rates of fitted trajectories for both example countries were multiplied by 0.75 and 1.25, respectively, at each time point.

[e] As a proportion of global PLHIV, the PrEP target for the France was 25000. This was multiplied by 2 given that the country was already close to that target.

[f] As a proportion of global PLHIV, the PrEP target for the USA was 157895. This was multiplied by 2 given that the country already surpassed that target.

[g] Only one example country with at least three years of PrEP user data was available for the SEARO region (Thailand). Viet Nam (WPRO) was selected as an additional model example. To generate six example trajectories, PrEP user growth rates of fitted trajectories for both example countries were multiplied by 0.75 and 1.25, respectively, at each time point.

[h] As a proportion of global PLHIV, the PrEP target for Australia was 3816. This was multiplied by 20 given that the country already surpassed that target by a factor of 10.

[i] Only two example countries with at least three years of PrEP user data were available for the WPRO region (Australia and Viet Nam). Thailand (SEARO) was selected as an additional example country.

Sensitivity analysis for the PrEP forecasting

As outlined above, a global PrEP equilibrium of 5 million PrEP users was used to estimate PrEP users in the example countries by 2030, which was necessary for fitting example curves. It did not constrain global projections, and, as shown in the main article, under one growth scenario there were more than 5 million PrEP users already in 2023. A sensitivity analysis was conducted in which the global PrEP equilibrium was set at 30% lower (3.5 million) and 30% higher (6.5 million). Figure S1 show the results of the forecasting. The means of the observed growth scenarios were within the range of scenarios presented in the main article and the mean was about 13% higher with the higher equilibrium assumption (Figure S1A) and about 19% lower with the lower equilibrium assumption (Figure S1B). With these different assumed equilibria, twelve additional PrEP trajectories were estimated that were higher or lower than the ones presented in the main article. This underscores the importance of considering the range of possible future outcomes and uncertainty around the estimates.

A: Global PrEP user forecast scenarios without COVID disruptions: 3.5 million equilbirum

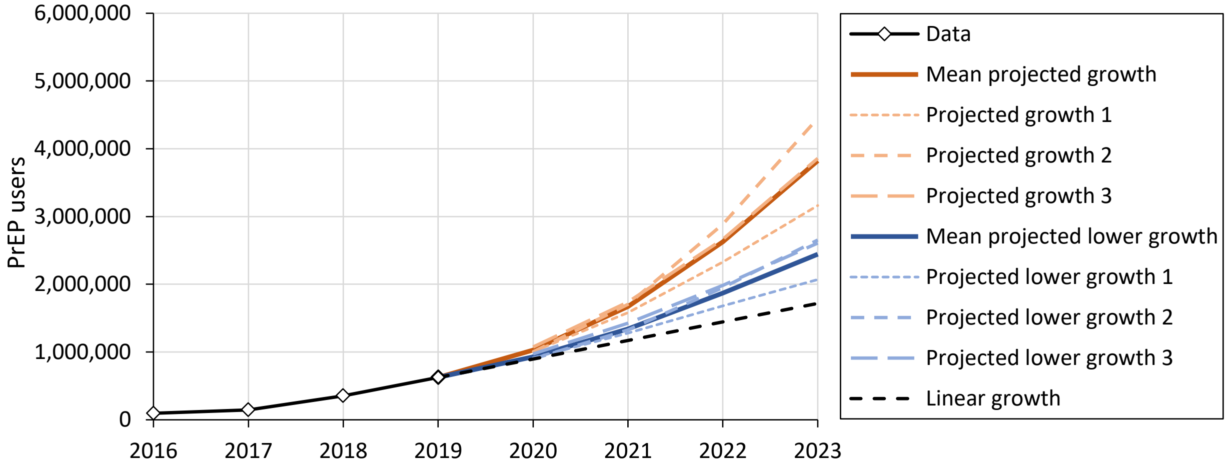

B: Global PrEP user forecast scenarios without COVID disruptions: 6.5 million equilbirum

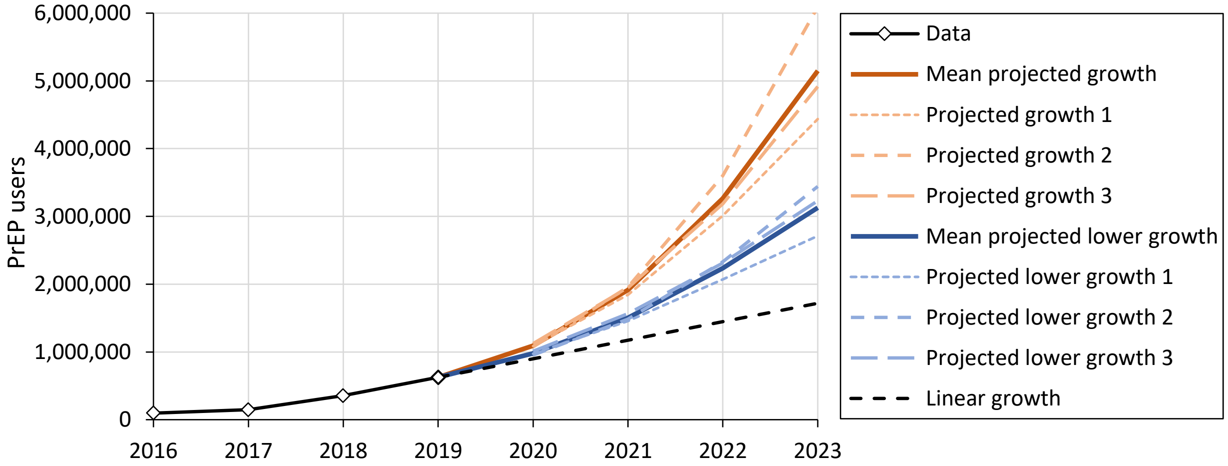

Figure S1: Forecasted numbers of people who have received oral pre-exposure prophylaxis (PrEP) at least once (PrEP users) globally per year until 2023 without assumed COVID-19 disruptions with an assumed global PrEP equilibrium for example trajectory fitting of 3.5 million (A) and 6.5 million (B). The projected growth (orange) was based on three observed example trajectories for each region applied to all countries in the corresponding region. The projected lower growth (blue) was based on the example trajectories with 25% lower growth at each time step. Countries that had adopted the WHO PrEP recommendations or were pending the adoption were assumed to introduce PrEP services in 2020. In the linear growth scenario, the same number of PrEP users was added to each country as observed between the last two years of data availability and no country was assumed to introduce PrEP services.

References for the Research in context panel

Balayan T, Begovac J, Skrzat-Klapaczyńska A, et al. Where are we with pre-exposure prophylaxis use in Central and Eastern Europe? Data from the Euroguidelines in Central and Eastern Europe (ECEE) Network Group. HIV Med 2021; 22: 67–72.

Haldar P, Reza-Paul S, Arokiam Daniel R, et al. A rapid review of pre-exposure prophylaxis for HIV in the Asia-Pacific region: recommendations for scale up and future directions. Sex Health 2021; 18: 31–40.

Hodges-Mameletzis I, Dalal S, Msimanga-Radebe B, Rodolph M, Baggaley R. Going global: the adoption of the World Health Organization's enabling recommendation on oral pre-exposure prophylaxis for HIV. Sex Health 2018; 15: 489–500.

Moseholm E, Gilleece Y, Collins B, et al. Achievements and gaps to provide pre-exposure prophylaxis (PrEP) for women across the European Region - Results from a European survey study. J Virus Erad 2020; 7: 100026.
